# Dietitians’ Attitudes and Understanding of the Promotion of Grains, Whole Grains, and Ultra-Processed Foods

**DOI:** 10.3390/nu14153026

**Published:** 2022-07-23

**Authors:** Natasha Krois, Jaimee Hughes, Sara Grafenauer

**Affiliations:** 1 School of Medical, Indigenous and Health Sciences, University of Wollongong, Northfields Avenue, Wollongong 2522, Australia; natasha.krois1@gmail.com; 2 Grains & Legumes Nutrition Council, 1 Rivett Rd, North Ryde 2113, Australia; jaimee.hughes2@gmail.com; 3 School of Health Science, Faculty of Medicine and Health, University of New South Wales, Randwick 2052, Australia

**Keywords:** whole grain, grains, ultra-processed, dietitian, education, NOVA

## Abstract

NOVA is a food-classification system based on four levels of processing, from minimally processed to ultra-processed foods (UPFs). Whole-grain-containing commercial breads and ready-to-eat breakfast cereals are considered ultra-processed within NOVA, despite being considered core foods in the Australian Dietary Guidelines. These food categories contribute the greatest quantities of whole grain in the Australian diet, although consumption is less than half of the 48 g/day daily target intake. Dietitians are key to disseminating messages about nutrition and health; therefore, an accurate understanding of whole grains and the effects of processing is critical to avoid the unnecessary exclusion of nutritionally beneficial foods. The aim was to utilise an online structured questionnaire to investigate dietitians’ attitudes to the promotion of grains and whole grains and understand their level of knowledge about and attitudes towards NOVA and the classification of specific whole-grain foods. Whole-grain foods were perceived positively and are regularly promoted in dietetic practice (*n* = 150). The dietitians tended not to consider whole-grain breads and ready-to-eat breakfast cereals as excessively processed, although most generally agreed with the classification system based on the extent of processing. If dietitians intend to incorporate NOVA and concepts of UPFs in their counselling advice, the anomalies regarding the categorisation of whole-grain choices and optimum intakes should be addressed.

## 1. Introduction

Consumers are faced with an ever-increasing array of products available to purchase, and there is considerable marketing of packaged and convenience foods. Most countries have developed food-based dietary guidelines to make it easier for consumers to choose healthier options. However, while it may be easier for consumers to judge the degree of healthfulness of single-ingredient food items, foods with multiple ingredients may pose a challenge and necessitate further interpretation [1]. There may be multiple mechanisms through which individuals make food choices; however, the dietitian’s role in educating and supporting individuals, groups, and populations to make healthful food choices has become increasingly important [1]. The responsibility of a dietitian to translate nutrition science into dietary patterns extends across a spectrum of domains, from individualised advice and public health settings through to working alongside food industries. Understanding the determinants that influence dietitians’ perceptions of foods and the impact this has on practice may be helpful in considering how anomalies in messaging around grain foods and processing might be managed, such as the classification of whole-grain breads and cereals as ultra-processed according to the NOVA food-classification system.

An increasingly widespread food-classification system termed NOVA was developed in Brazil by Monteiro et al. (2016) [2]. The system has been integrated into the Brazilian Dietary Guidelines, and directs consumer choice by grouping food products into four main categories based on their degree of processing [3]. According to recent research, a higher consumption of ultra-processed foods is associated with a higher risk of developing chronic diseases, such as cardiovascular disease, type 2 diabetes, and obesity [4]. Monteiro et al. also argued that the classification of foods by their nutritional composition and origin—as in the Australian Dietary Guidelines (ADG) [5]—is a lesser indicator of the relationship between food and health than classification by the extent of food processing [6]. The four categories are as follows: (1) unprocessed and minimally processed foods, (2) processed culinary ingredients, (3) processed foods, and (4) ultra-processed foods (UPFs). NOVA researchers describe UPFs as, typically, energy-dense foods of poor nutritional quality that are low in dietary fibre and contain excessive amounts of sodium, simple sugars, and saturated and trans fats [7], and recommended their avoidance [3]. When NOVA and the ADGs are compared, many of their overall dietary messages are similar; however, discrepancies between the two systems exist [8]. An analysis of the 2011–12 National Nutrition and Physical Activity Survey (NNPAS) indicated that 23.5% of core foods (foods that should form the basis of a healthy diet [9]) were classified as ultra-processed, and 31.2% of discretionary foods were classified as not ultra-processed [8]. Notably, NOVA categorises core foods, such as mass-produced packaged breads and buns, as well as ready-to-eat breakfast cereals, as UPFs; however, discretionary choices, such as butter, cream, sugar, honey, homemade cakes, and biscuits were not classified within this category [8,10]. This is important, as breads, bread rolls, and ready-to-eat breakfast cereals are the largest contributors to Australian’s whole-grain intake [11]. Therefore, to the advice to avoid these foods, as they are classified as UPFs, is likely to decrease whole-grain consumption. As a study by Monteiro et al. (2019) states: “Processes and ingredients used to manufacture ultra-processed foods are designed to create highly profitable (low-cost ingredients, long shelf-life, emphatic branding), convenient (ready-to-consume), hyper-palatable products liable to displace all other NOVA food groups, notably unprocessed or minimally processed foods” [12]. However, this description does not consider the nutrient content of the foods, or the related health-outcome research, and implies that convenient and packaged food products are automatically classified as ultra-processed without further examination through translational research.

Evidence suggests that consumers’ ability to accurately interpret nutrition information is poor, particularly in relation to bread choices [13]. This accentuates the responsibility of dietitians, particularly in public-health and food-industry roles, to advocate for products to be clearly labelled, and for nutrition information provided to be unambiguous. Dietitians also have a responsibility to distinguish misconceptions from evidence-based nutrition and assist consumers in obtaining reliable information to inform their dietary decisions. Those working in the broader fields of public health or industry also have a responsibility to advocate for the distribution of health messages and resources that provide accurate and reliable information. If dietitians are to be viewed as informed and credible sources, then they are required to have a sound understanding of nutrients, foods, and a range of dietary concepts, which includes an understanding of food processing, its effects on health, and the established links with disease for individuals, sub-groups, and populations. Dietitians can also provide insight into the barriers that consumers may face in implementing dietary advice; thus, research on dietitians’ understanding is a valuable method to inform health-promotion strategies and educate health professionals to provide support accordingly.

The Theory of Planned Behaviour (TPB) is a behavioural model that can be applied to understand and predict an individual’s actions and intentions. In relation to this study, the TPB suggests that the intent of a dietitian to promote and/or exclude grains, whole grains, and the NOVA food classification system is influenced by personal attitudes, subjective normative beliefs, and perceived behavioural control [14]. Subjective normative beliefs are a key constituent of the TPB, implying that a dietitian’s intent to promote whole grains may be influenced by the perception of whole-grain promotion by other dietitians and behaviours that they take to be normative [15]. A study conducted by Chase et al. (2003), prior to the release of NOVA, identified that subjective normative beliefs were the greatest predictors of dietitians’ intent to promote whole-grain foods, since they were 11.9 times more important than attitudes and 2.3 times more important than perceived behavioural control [14]. Therefore, the aim of this study was to utilise an online structured questionnaire to investigate dietitians’ attitudes to the promotion of grains and whole grains and understand the extent of dieticians’ knowledge of and attitudes towards NOVA and the classification of specific whole-grain foods. A further aim was to examine dietitians’ familiarity with the NOVA food-classification system and their knowledge and attitudes towards the classification of specific foods within this system, particularly those that contain whole grains.

## 2. Materials and Methods

### 2.1. Survey Design

The Qualtrics XM Platform ™ (Provo, UT, USA) was utilised to distribute an anonymous online structured questionnaire [16] targeting dietitians from a number of countries, including Australia, New Zealand (NZ), Canada, the United States (US), United Kingdom (UK), and South Africa between April 2021 and July 2021. Survey questions were pilot-tested in consultation with stakeholders, including dietitians, to test construct and content validity. The final 10 min survey consisted of 41 questions and utilised an open and closed questionnaire design with free text and multiple-choice responses, Likert scales, matrix, and rank-order questions (Supplementary Materials File S1). The survey was divided into four parts: (1) demographic questions, including age, gender, level of education, self-reported dietetic credentials, country where dietetics education program was completed, and main area of dietetic practice (2, 3) advice that survey respondents might provide in one-on-one consultations, in group sessions, or via media about grain foods and whole-grain foods (4), and questions related to the NOVA food-classification system (Supplementary Materials File S2). The TPB was incorporated into the survey design and was utilised to propose questions aimed at drawing out the key factors that may influence dietitians’ perceptions and the integration of grains, whole grains and/or ultra-processing in their normal practice advice (Supplementary Materials File S2). Questions were assigned to one or more of the three constituents of the TPB—attitudes, subjective normative beliefs, and perceived behavioural control—to systematically investigate dietitians’ understanding and perceptions of and attitudes to grains, processing, and the NOVA food-classification system, how these were influenced, and how this translated into practice (Supplementary Materials File S2). The attitudes were investigated in questions regarding the promotion of grains, including sources, which were classified as ultra-processed, specifically aiming to explore whether any particular grain foods were considered excessively processed, as well as underlying reasons for limiting grain consumption. Subjective normative beliefs were investigated in questions exploring participants’ perceptions of the understanding and prioritisation of whole grains by other dietitian colleagues. Perceived behavioural control was investigated in questions exploring participant confidence and the frequency with which advice about whole grains and NOVA in practice was provided. Ethical approval for this study was obtained from the University of Wollongong Human Research Ethics Committee (HREC), approval number 2021/038.

### 2.2. Participants

This study employed voluntary-response sampling, including a combination of convenience, snowballing, and purposive sampling, as it is a relatively fast and inexpensive means of response collection. Dietitians within several countries—Australia, NZ, Canada, US, UK, and South Africa—were recruited to participate in the study. The countries were chosen based on similar dietetic education systems and, to some extent, similar dietary patterns. Participant recruitment was achieved in several ways, as follows: online advertisement on Grains & Legumes Nutrition Council (GLNC); social media platforms, including LinkedIn, Facebook, Twitter, and Instagram; GLNC and Dietitians Australia (DA); and e-newsletters, including those published by and e-newsletters international dietetic networks, such as the organisations, Oldways (US), Dietitian Connection (Australia), and Education in Nutrition (Australia). To encourage participation, eligible participants were offered the opportunity to enter their email in a separate survey to be placed into a draw to win one of six Portion Perfection gift cards at a value of AUD 100 for professional materials. Inclusion criteria required participants to be over the age of eighteen, a registered dietitian (RD), or an accredited practising dietitian (APD) with access to the internet and online technologies, such as a computer or smart phone, to undertake the questionnaire.

### 2.3. Response Analysis

Participant response data were exported from Qualtrics (Provo, UT, USA) to a Microsoft Excel™ spreadsheet (Version 16.53, Washington, DC, USA) for data analysis. Using Microsoft Excel™ (Version 16.53, Washington, DC, USA), descriptive statistics were applied for analysis of quantitative data. Content analysis was conducted for qualitative data from free-text responses, in which responses were assigned to reoccurring themes by the researcher (NK). A summary report of free-text responses was also generated by Qualtrics (Provo, UT, USA) to assist with the qualitative data analysis.

## 3. Results

A total of 199 respondents attempted the survey, of which 123 completed the survey in full and 76 provided partial responses. However, of the 199 respondents, three participants did not meet the inclusion criteria, as they did not hold appropriate dietetics credentials, and 46 participants did not respond to the questions beyond those in the demographic characteristics, which left a total 150 responses included in the analysis (122 full and 28 partial responses).

### 3.1. Demographics

The number of participants who had completed their dietetics education in Australia was far greater than the number of participants who completed education elsewhere, accounting for 68% of the total number of participants (Table 1). Therefore, we compared Australia to other countries. However, as the initial analysis showed minimal variation in the responses, no further analysis by country was undertaken. Similarly, the analysis of the responses by age category indicated minimal variation between the groups, and in limited instances of dissimilarity, large disparities in the number of participants between age categories were evident, such as 25–34 years (*n* = 51) versus 65+ years (*n* = 8), limiting the value of the comparisons. The number of participants in each age category was compared to the age profiles of the current Dietitians Australia members, identifying the greatest number of dietitians in both instances to be in the 25–34-years category.

### 3.2. Perceived Value, Attitudes, and Recommendations of Grains, including Whole Grains

Grain foods, specifically whole-grain varieties, were perceived positively by the dietitians, and are regularly promoted in advice (Table 2). The participants frequently recommended whole grains (134/150) and high-fibre grains (114/150). This is in line with the fact that most of the participants (102/150) encouraged the consumption of grain foods based on national dietary guidelines, such as the Australian Dietary Guidelines (Table 2).

On the other hand, some dietitians specifically stated they did not recommend refined or non-whole -grain foods (57/150). When recommending whole-grain foods in practice, the dietitians most commonly used a specific suggestion (e.g., swap refined ready-to-eat cereal for oats; swap white for wholemeal/whole-grain bread), promoting the substitution of refined grains with whole grain varieties. Notably, the specific grain-based foods most frequently recommended by the dietitians were bread (132/150) and breakfast cereal (115/150).

Generally, the dietitians were aware of the benefits of whole-grain consumption; however, some of these benefits were poorly recognised (Table 3). Almost all the dietitians were familiar with the benefits associated with the high fibre content of whole grains (144/149), as well as their benefits for blood-glucose control (133/149). However, some of the benefits of whole grains for cardiovascular health were more poorly recognised in comparison (Table 3). The prioritisation of whole grains for weight control (81/149) and their potential for reducing inflammation (62/149) were also less well known. The participants recognised suitable contraindications of whole grains (e.g., gluten containing grains in coeliac disease) in some instances (87/149).

**Figure 1:**
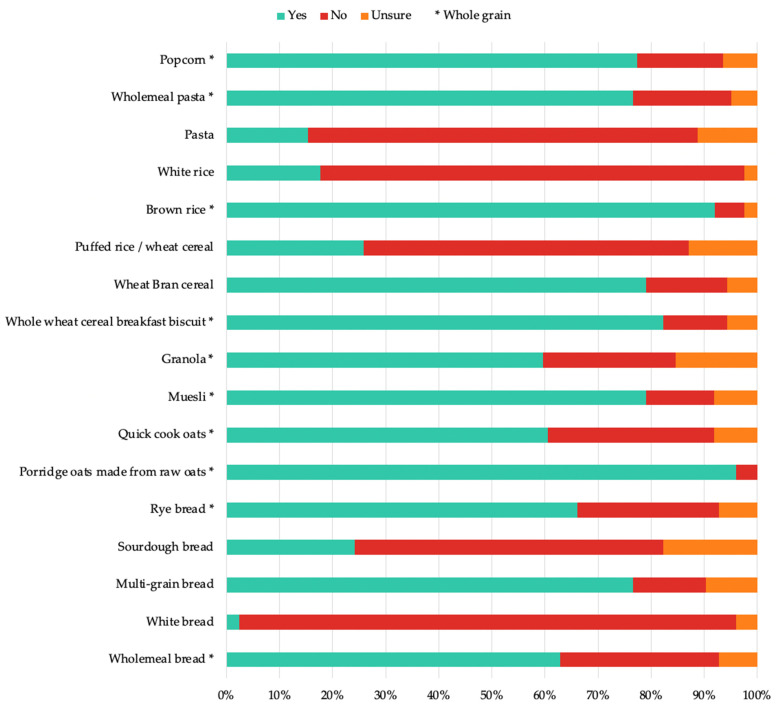
Identification of whole-grain- and non-whole-grain-containing foods by dietitians. *: indicates that the food is whole-grain.

When the participants were asked to identify whole-grain sources by responding ‘yes’, ‘no’, or ‘unsure’ to a list of whole-grain- and non-whole-grain-containing foods, the majority of the responses showed a good knowledge of whole grains (Figure 1). However, the categorization of some foods showed a poor understanding; for example, some dietitians (39/124) presumed that quick cook-oats would not be whole-grain and that multi-grain bread and wheat-bran cereal were whole-grain (95/124 and 98/124, respectively). The dietitians generally recognised refined foods as non-whole-grain, such as white bread (116/124).

The majority of the dietitians were confident in providing whole-grain education (with 137/149 scoring 4 or 5, with 5 being the most confident) and agreed (130/142 claimed to agree or strongly agree) that dietitians are well educated about the importance and benefits associated with whole-grain-food consumption. In a question assessing the perception of whole-grain promotion by other dietitians, most of the dietitians (90/142) believed that other dietitians regularly promote and prioritise the intake of whole-grain foods, and 39/142 believe this occurs ‘somewhat’. The dietitians suggested that whole-grain education for dietitians could be improved by ‘better resources for clients’ (102/142), ‘CPD/online learning’ (90/142), ‘better resources from dietary guidelines/national policy’ (70/142), and ‘marketing campaigns’ (41/142).

### 3.3. Perceived Barriers to Whole-Grain Consumption

**Figure 2:**
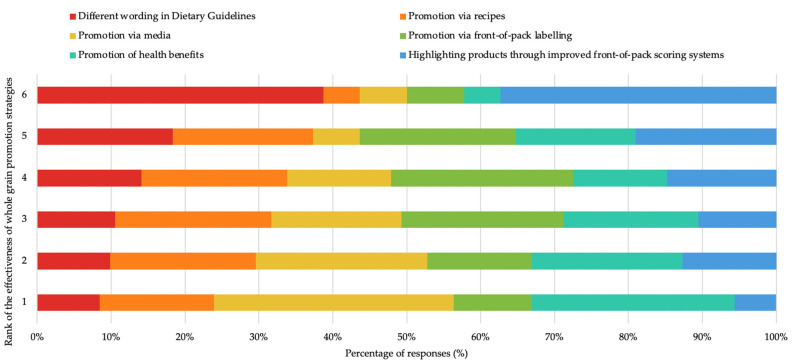
Dietitians’ responses ranking the effectiveness of previously used whole-grain-promotion strategies on a scale of 1–6 (1 being most effective, 6 being least effective).

The dietitians perceived there to be several key barriers to whole-grain intake. Alongside this, the majority were not confident that the public is well educated on the importance and benefits associated with whole-grain-food consumption (86/142). Some were ambivalent (43/142), and only thirteen thought the public was well educated. The barriers to whole-grain consumption cited by the dietitians frequently related to ‘taste’ (83/116) and ‘concerns about carbohydrate intake’ (66/116) (Table 4). When asked which strategies could help to overcome the barriers to whole-grain consumption, several themes were identified across the responses, which were most commonly related to education (86/116) (Table 4). The participants were asked to rank the strategies they had previously used to improve whole-grain intake (on a scale of 1–6, with 1 being most effective and 6 being the least effective) (Figure 2). Promotion via the media and the promotion of the health benefits were perceived as the two most effective strategies. Improved front-of-pack scoring systems and changes to dietary guidelines were ranked as the least effective.

### 3.4. Knowledge, Attitudes and Use of the NOVA Classification System

**Figure 3:**
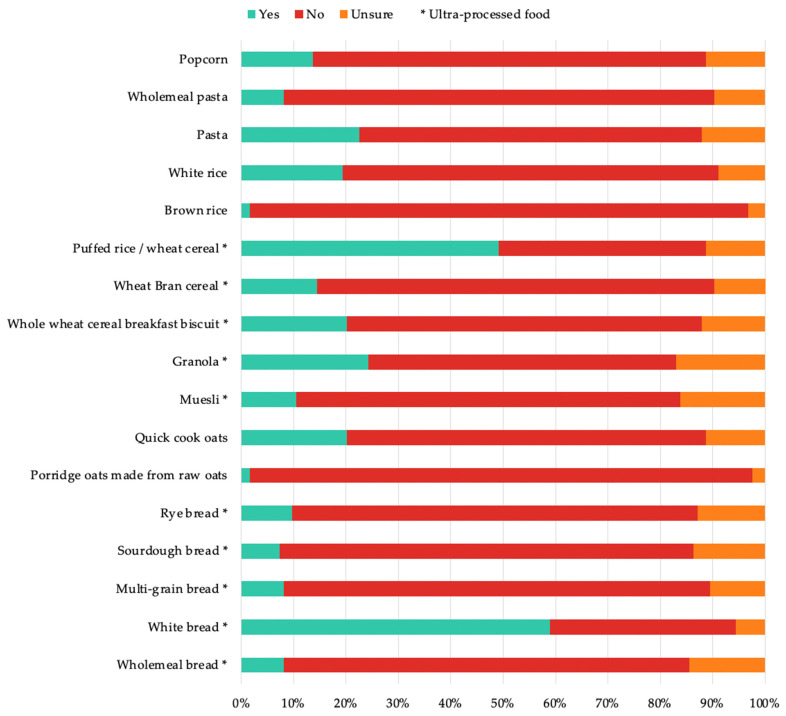
Identification of ultra-processed (NOVA classifications) and non-ultra-processed grain foods by dietitians. A star indicates that a food is classified as ultra-processed.

Many of the dietitians (75/124) were unfamiliar with the NOVA classification system, although the majority (116/124) were familiar with the advice to limit the intake of highly processed/ultra-processed foods. When asked if they incorporated and/or referred to NOVA or the processing of food in practice, the results were mixed, with 18/124 dietitians reporting that they ‘’always’ referred to NOVA, 29/124 reporting that they referred to NOVA ‘most of the time’, 9/124 claiming to do so ‘about half of the time’, 18/124 claiming to do so ‘sometimes’, and 50/124 claiming to never refer to NOVA in practice. Furthermore, many of the dietitians (75/124) agreed (agree or strongly agree) with NOVA’s classification of foods, while 45/124 were ambivalent and 4/124 disagreed. The participants were asked to elaborate on why they responded in the manner that they did, and a content analysis was undertaken to categorise the responses. Many of the reported reasons related to unfamiliarity with NOVA (45/124), but some also related to support for a food-classification system based on processing (32/124). Moreover, when dietitians were asked to identify which grain foods are classified as ultra-processed, most dietitians considered whole grain foods to not fit into this classification, whereas their refined counterparts were generally considered to be ultra-processed (Figure 3). For example, wholemeal bread and white bread were considered as UPFs by 10/124 and 73/124, respectively. 

The dietitians’ attitudes to and perceptions of whole grains, NOVA, and UPFs were further explored in a matrix-style question prompting the participants to indicate the extent to which they agreed or disagreed with the listed statements (Table 5). The majority (92/123) of the dietitians agreed (agreeing or strongly agreeing) that UPFs should generally be avoided; however, the whole-grain breads and cereals should not be included in this classification. Furthermore, 47/123 of the dietitians agreed (agreeing or strongly agreeing) that they were less inclined to recommend the avoidance of UPFs, knowing that this classification may include whole-grain breads and cereals. On the other hand, some dietitians (30/123) agreed (agreeing or strongly agreeing) that knowledge of the classification of these grain foods as ultra-processed had negatively affected their perception of these sources of whole grains, and 16/123 agreed (agreeing or strongly agreeing) that they were less inclined to recommend these foods in practice.

## 4. Discussion

Grains, especially whole grains, were perceived positively by the dietitians and were regularly promoted in their advice. Encouragingly, almost all the dietitians recommended grain foods in consultation, group sessions and/or through media messages, and, more specifically, recommended whole-grain varieties over refined-grain options. One quarter of the dietitians did not prioritise grain foods in advice for general healthy eating, suggesting that while the majority of the dietitians were aware of the role of grains in a healthy dietary pattern, others may have prioritised other foods or food groups in their practice. A study by Chase et al. (2003) reported that dietitians perceive the prioritisation of other dietary changes, a lack of time, and a lack of resources to use with clients to be barriers to whole-grain promotion [14]. Furthermore, the majority of the dietitians participating were confident in their provision of whole-grain education (137/149) and agreed that dietitians were well educated regarding the importance and benefits associated with whole-grain-food consumption. This was positive, as the TPB implied that an individual’s perceived behavioural control, including feelings of self-efficacy, influence the dietitian’s intent to promote whole grains [17]. As confidence increases, whole-grain promotion is more likely to occur. The dietitians advised that whole-grain education could be improved for dietitians through better resources for clients, CPD/online learning, and better resources from dietary guidelines/national policy, indicating that the dietitians may have perceived the current quality of these resources to be insufficient for optimal learning. Future education strategies for dietitians may benefit from targeting these areas.

The dietitians tended to be aware of and refer to national dietary guidelines (NDG) to inform whole-grain advice. NDGs are important educational tools, translating the complex matrix of nutrition science into simple messages that enable consumers to make positive food choices and form healthy dietary patterns [18]. Country-specific guidelines exist worldwide, incorporating positive messages about grains, especially whole grains. For example, the US Dietary Guidelines recommend that ‘at least half of total grains should be whole grains’, while the UK Dietary Guidelines recommend choosing ‘whole grain or higher fibre versions with less added fat, salt and sugar’ and, similarly, the ADG recommend choosing ‘mostly whole grain and/or high cereal fibre varieties’ [5,19,20]. These results indicated that NDGs are meaningful resources that influence dietitians’ understanding and recommendations in practice. Interestingly, the participants did not report that any other wording in the dietary guidelines would specifically further encourage consumer intake of whole grains and appeared satisfied with the current guidelines.

Generally, the dietitians displayed a good level of knowledge when identifying whole grains; however, some items were less well understood. For example, many of the dietitians were able to correctly identify cereals such as raw oats and muesli as whole grains, although some incorrectly perceived that quick cooking may be a factor that differentiates whole-grain content. This suggests that dietitians may perceive whole grains that have undergone any processing, including the addition of other ingredients, to have inferior properties. It is true that processing may mean a product is not whole grain, but when these components (bran, germ, and endosperm) are included in the same proportion as the original grain, this constitutes a whole grain, and includes wholemeal. In this study, the dietitians appeared to be less informed of the nuanced information relating to what defines a whole grain or whole-grain food. Correspondingly, a study by Botelho et al. (2018) concluded that the nutritional quality of a product is associated with the product formulation, rather than the degree of processing [21]. Furthermore, many dietitians incorrectly presumed that wheat-bran cereal and multi-grain bread were whole grains. However, it is also notable that bran cereals are high in cereal fibre, one of the elements of whole grains that confer the most health effects [22]. Comparatively, a study by Chase et al. (2003) reported that dietitians’ ability to identify whole-grain products were low, with only 60% of dietitians correctly identifying whole-grain products according to a corresponding sample food label [14]. It is clear that the dietitians in this survey appeared to have a greater knowledge of what constitutes a whole-grain food, indicating that whole-grain education for dietitians may have improved over time.

Although more than half of the dietitians in this survey were unfamiliar with NOVA, the majority agreed with NOVA’s classification of foods based on the brief statement that the foods in this system are classified based on the extent of their processing. A study by Sadler et al. (2022) highlighted the ambiguities associated with concepts of processing, (ultra) processed food, and healthy food [23]. For example, an exploration of health professionals’ views of processed foods identified tensions between the concept of ‘food processing’, which was recognised as necessary for food sustainability and security, and that of ‘processed foods’ which was perceived as less healthy or natural [23]. Whilst the participants agreed that these are broad concepts that require differentiation, it was highlighted that consumers tend to view foods through a dichotomous classification of ‘good’ or ‘bad’, and that when they refer to so-called ‘processed foods’, they do not refer to the degree to which a food is processed but, rather, to their perception of its healthfulness [23]. Unsurprisingly, the dietitians generally agreed that the more a food is processed, the less favourable it may be for consumption. In line with this, a recent study demonstrated that a higher percentage of energy from UPFs was inversely associated with diet quality when applied to the ADG features of a healthy diet (for example, enjoying a variety of foods from each of the core food groups) [24]. Consequently, it is likely that as UPF consumption increases and whole-grain consumption decreases, reinforcing the divergence from concepts of healthy eating, as defined by the ADG.

Although the dietitians were familiar with the advice to limit the intake of highly processed/UPFs, it appeared that they demonstrated a knowledge gap in the identification of whole-grain foods that are classified as ultra-processed. For example, the dietitians in the survey were mostly unaware that commercial breads, including whole-grain varieties and ready-to-eat breakfast cereals, were included in the classification of ultra-processed. This may have been the result of a predilection towards the NOVA food-classification system, potentially overlooking the anomalies between the benefits of whole-grain consumption and the processing characteristics defined by NOVA. Generally, the dietitians associated UPFs with discretionary choices, such as those containing nutrients unfavourable to health, including added sugars, salt, or saturated fat Where dietitians perceived grain foods may apply to this category, these tended to be refined varieties, for example white bread, highlighting that this may be a differentiating factor in the participant’s identification of UPFs. Whole-grain foods were often considered to not be ultra-processed; for example, wholemeal bread and muesli were incorrectly identified by the majority of the dietitians as not ultra-processed, suggesting that dietitians do not associate whole grains with UPFs. Research has demonstrated that replacing refined grains with whole grains reduces the risk of cardiometabolic disease [25]. Research has also found nutrient-dense UPFs, including whole-grain breads and ready-to-eat breakfast cereals, to contribute to greater dietary quality [26,27], and that the message that they are to be ‘avoided’ may not be appropriate.

While it was encouraging that the majority of the dietitians remained positive towards sources of whole grains, worryingly, a small number (16/123) became dubious about advising their consumption, when alerted to the potential for these to be classified as ultra-processed. Further education may be necessary to guide dietitians in promoting the avoidance of UPFs, to ensure that they are well informed on the whole-grain-food sources included in this classification and the consequences of their avoidance. A study assessing the factors that influence Australian dietitians’ perceptions of packaged foods (*n* = 117) reported that alongside ingredients and nutrition composition, the shelf-life and storage of packaged food items were indicators of product healthfulness, taking into consideration the potential of a food item to cause food-borne illness [1]. This is notable given that the addition of additives, which enhance shelf stability, results in the classification of ultra-processed according to the NOVA food classification system, despite the benefits of additives for food safety. It is essential that dietitians recognise the factors that influence individual and population eating practices, such as accessibility, time, income, and skills [28]. For example, NOVA classifies commercial breads as ultra-processed, but not homemade breads. However, it is questionable whether it is feasible for individuals to make their own bread, and whether doing so provides worthwhile nutritional and health benefits compared with purchasing a commercial loaf. The findings of this study provide the basis for the reclassification of whole-grain breads and ready-to-eat breakfast cereals within NOVA, utilising evidence-based health-outcome data to reevaluate the classification rules.

### Limitations

The findings of this survey should be considered in light of its limitations. This study used voluntary response sampling rather than randomised sampling; therefore, it is unlikely that the results are representative of the dietetic profession as a whole. It is also possible that the distribution of the survey link via the GLNC social media channels attracted nutrition professionals that have an interest in or prior knowledge of grains, whole grains, and the anomalies of the NOVA classification system in relation to whole-grain foods, as previously mentioned. It is known that some individuals may be inherently more likely to participate than others; for example, those who respond may have stronger opinions or be more invested in the subject matter than those who do not, further limiting the generalisability of the results to the profession as a whole. The small number of responses from the participants from selected countries outside of Australia limited the ability to conduct a statistical analysis to compare differences between countries.

## 5. Conclusions

Although the findings are not representative of the entire dietetic profession, whole-grain foods were perceived positively by the dietitians who were included in the survey, who reported regularly promoting them in their practice. The dietitians appeared confident in their ability to provide whole-grain education and believed they were well educated about whole grains. This is important, as dietitians are key health professionals in the dissemination of messages about nutrition and health and the provision of specific food advice. The dietitians generally had a good ability to identify whole grains and their health benefits, although there were some gaps in this knowledge and some errors. Further education may be required in these areas. The dietitians acknowledged that the public may not be well educated as to the benefits of whole grains, and they perceived several barriers to whole-grain consumption. The dietitians believed that improving knowledge of the health benefits of whole grains and their promotion via the media have been the most effective methods for their promotion, indicating approaches for future promotional strategies.

The dietitians tended to use NDG to inform their whole-grain advice. However, if NOVA were to be more widely promoted, given that it classifies whole-grain breads and ready-to-eat breakfast cereals as ultra-processed, these valuable sources of whole grain may be discouraged. However, the majority of the dietitians did not consider these sources as ultra-processed and remained positive towards recommending these foods in dietary advice. Rather, the dietitians tended to associate UPFs with discretionary foods and foods containing nutrients that negatively affect health, although they generally considered a system based on the degree of processing as an effective way to categorise foods. If dietitians are to refer to and incorporate NOVA and concepts of UPFs in advice, the anomalies between messages promoting the avoidance of UPFs and messages promoting whole-grain intake must be addressed.

## Figures and Tables

**Table 1:** Demographic characteristics of participants.

Demographic Characteristics	Count (%)
**Gender**	
Female	142 (94.7)
Male	6 (4.0)
Prefer not to answer	2 (1.3)
Prefer to self-describe	0 (0)
**Age**	
18–24	19 (12.7)
25–34	51 (34.0)
35–44	31 (20.7)
45–54	25 (16.7)
55–64	16 (10.7)
65+	8 (5.3)
**Level of Education**	
Certificate/diploma	3 (2.0)
Bachelor degree	64 (42.7)
Masters degree	77 (51.3)
Ph.D.	6 (4.0)
**Dietetic credential**	
Accredited practicing dietitian	110 (73.3)
Registered dietitian	35 (23.3)
Qualified dietitian but not registered	3 (2.0)
Other	2 (1.3)
**Country dietetics education was completed**	
Australia	102 (68.0)
New Zealand	5 (3.3)
Canada	3 (2.0)
United States	33 (22.0)
United Kingdom	1 (0.7)
South Africa	4 (2.7)
Other	2 (1.3)
**Country currently practising as a dietitian**	
Australia	110 (73.3)
New Zealand	2 (1.3)
Canada	0 (0.0)
United States	30 (20.0)
United Kingdom	2 (1.3)
South Africa	2 (1.3)
Other	4 (2.7)
**Years practised as a dietitian**	
≤5 years	50 (33.3)
6–10 years	38 (25.3)
11–20 years	24 (16.0)
>20 years	38 (25.3)
**Main area of work**	
Community/public health	31 (20.7)
Food service	3 (2.0)
Academia/education	8 (5.3)
Research	4 (2.7)
Clinical (hospital)	32 (21.3)
Clinical (primary care)	13 (8.7)
Private practice	38 (25.3)
Corporate nutrition	4 (2.7)
Food industry	3 (2.0)
Retail	3 (2.0)
Other	11 (7.3)

**Table 2:** Dietitian recommendations, sources and frequency of advice related to grains and whole grains.

Question	Response	Count (%)
Do you recommend or discuss grain foods in consultation, groups sessions or via media messages?	Yes No	149 (99.3) 1 (0.7)
Are grain foods prioritised in your advice for general healthy eating?	Yes No Other	116 (77.3) 19 (12.7) 15 (10.0)
Do you promote amounts of grain foods based on National Dietary Guidelines?	Yes No	121 (80.7) 29 (19.3)
Do you recommend whole grain foods?	Yes No	148 (99.3) 1 (0.7)
Considering your advice on general healthy eating, how often do you recommend whole grain foods in dietetic practice?	Always Most of the time About half of the time Sometimes Never	74 (49.7) 63 (42.3) 6 (4.0) 6 (4.0) 0 (0.0)
What sources of information do you most often use for your advice relating to whole grain food intake? *	National Dietary Guidelines Government Resources Resources from professional organisations Resources from non-government organisations Other	102 (68.5) 26 (17.5) 82 (55.0) 44 (29.5) 11 (7.4)

* Question allowed respondents to select more than one answer; consequently, values presented are the proportion of respondents selecting each point.

**Table 3:** Dietitians’ understanding of the benefits of whole grains and situations in which they should be prioritised.

Question	Response	Count (%)
In your opinion, what are the nutrition and health benefits of whole grain foods? *	High fibre	144 (96.6)
	Low GI	120 (80.5)
	Improves weight control	104 (69.8)
	Improves blood glucose control	133 (89.3)
	Reduces insulin resistance	78 (52.4)
	Increases HDL-cholesterol	40 (26.9)
	Decreases LDL-cholesterol	95 (63.8)
	Lowers blood pressure	51 (34.2)
	Reduces inflammation	62 (41.6)
	Reduces risk of heart disease	102 (68.5)
	Management and reduced risk of type 2 diabetes	112 (75.2)
	Protective factor against colorectal cancer	119 (79.9)
	Other	18 (12.1)
Typically, in what situations might you prioritise whole grain foods in dietetic practice? *	I do not prioritise whole-grain foods in practice	1 (0.7)
	In general dietary advice	112 (75.2)
	To increase dietary fibre intake	130 (87.3)
	For weight control	81 (54.4)
	For diabetes management	106 (71.1)
	For blood-glucose control	105 (70.5)
	For cholesterol management	99 (66.4)
	For blood-pressure management	43 (29.9)
	Other	8 (5.4)
Are there any reasons why you would not recommend whole grain foods to a patient/client/group?	Contraindicated	87 (56.9)
	No reasons	46 (30.1)
	Individual taste preferences	10 (6.5)
	Low-carbohydrate diet	5 (3.3)
	Other client priorities	2 (1.3)
	Weight loss	2 (1.3)

* Question allowed respondents to select more than one answer; consequently values presented are the proportion of respondents selecting each point.

**Table 4:** Perceived barriers to whole-grain intake, and suggested strategies to overcome them.

Question	Response	Count (%)
In your opinion, what are the barriers to whole grain food consumption? *	Taste	83 (71.6)
	Concerns about carbohydrate intake	66 (56.9)
	Culinary skills (e.g., easy recipes)	65 (56.0)
	Time taken to prepare	52 (44.8)
	Price	46 (39.7)
	Other (please specify)	37 (31.9)
	Availability	26 (22.4)
	There are no barriers	2 (1.7)
What strategies could help overcome the barriers to whole grain consumption?	Education	86 (51.5)
	Public-health-promotion messages	31 (18.6)
	Food-industry action	24 (14.4)
	Improving individual acceptability	22 (13.2)
	Using evidence-based practice	2 (1.2)
	Not sure	2 (1.2)

* Question allowed respondents to select more than one answer; consequently, values presented are the proportion of respondents selecting each point.

**Table 5:** Dietitians’ attitudes to and perceptions of whole grains, NOVA, and UPFs.

Statement	Count (%)			
	Disagree	Neither Agree-Nor Disagree	Agree	Not Sure
I agree with the classification in NOVA for breads as “ultra-processed foods” if they are packaged and fortified.	75 (61.0)	16 (13.0)	29 (23.6)	3 (2.4)
I agree with the classification in NOVA for ready-to-eat breakfast cereals as “ultra-processed foods” even if they are fortified.	62 (50.4)	23 (18.7)	34 (27.6)	4 (3.3)
Knowing that some whole grain breads and ready-to-eat cereals are classified as “ultra-processed foods” has negatively impacted my perception of these sources of whole grains.	63 (51.2)	28 (22.8)	30 (24.4)	2 (1.6)
I am less inclined to recommend whole grain breads and ready-to-eat cereals in dietetic practice knowing that they are classified as “ultra-processed foods”.	83 (67.5)	21 (17.1)	16 (13.0)	3 (2.4)
I am less inclined to recommend avoidance of ultra-processed foods knowing that they may include some whole grain foods (such as some ready-to-eat cereals and breads).	43 (35.0)	29 (23.6)	47 (38.2)	4 (3.3)
I generally agree to avoid ultra-processed foods but do not agree that whole grain breads and cereals should be included in this classification.	10 (8.1)	16 (13.0)	92 (74.8)	5 (4.1)

## Data Availability

Not applicable.

## Supplementary Materials

The following supporting information can be downloaded at: https://www.mdpi.com/article/10.3390/nu14153026/s1, Supplementary Materials File S1: Survey Questions; Supplementary Materials File S2: Application of survey questions relative to the constituents of the Theory of Planned Behaviour.
