# Supplementary material for: Dietitians’ Attitudes and Understanding of the Promotion of Grains, Whole Grains, and Ultra-Processed Foods
Source: Nutrients. 2022 Jul 23;14(15):3026. doi: 10.3390/nu14153026 (PMC9331638; doi:10.3390/nu14153026)
Supplement: Supplementary file 1 [file nutrients-14-03026-s001.zip › Supplementary Materials 1 Survey Questions.pdf]

Supplementary Materials

Survey Questions

1. What is your age group?
   - 18-24 years
   - 25-34 years
   - 35-44 years
   - 45-54 years
   - 55-64 years
   - 65 years or older
2. What is your gender?
   - Female
   - Male
   - Prefer not to answer
   - Prefer to self-describe: ____________________
3. What is your highest level of education that you have completed?
   - Certificate (non-trade) or Diploma
   - Bachelor Degree (including Honours)
   - Masters Degree
   - PhD
4. What is your Dietetic credential?
   - Accredited Practising Dietitian (APD)
   - Registered Dietitian (RD)
   - Qualified Dietitian but not registered.
   - Other (please specify):
5. In what country did you complete your dietetics education program?
   - Australia
   - New Zealand
   - Canada
   - US
   - UK
   - South Africa
   - Other (please specify): ____________________
6. In which country are you currently practising as a dietitian?
   - Australia
   - New Zealand
   - Canada
   - US
   - UK
   - South Africa
   - Other (please specify): ____________________
7. How many years have you practised as a dietitian?
   - ≤5 years
   - 6-10 years
   - 11-20 years
   - >20 years
8. What is your **main** area of Dietetic Practice?
   - Community/public health
   - Food service
   - Academia/education
   - Research
   - Clinical (hospital)
   - Clinical (primary care)
   - Private practice

- Corporate Nutrition
- Food Industry
- Retail
- Other (please specify): ___________________

Grains

This survey is about advice you might provide in one-on-one consultations, in group sessions or via media (including social media) about grain foods.

9. Do you recommend or discuss grain foods in consultations, group sessions or via media messages?
   - Yes
   - No
10. Do you promote amounts of grain foods based on National Dietary Guidelines?
   - Yes
   - No
11. Are grain foods prioritised in your advice for general healthy eating?
   - Yes
   - No
   - Other (please specify): ___________________
12. Considering your advice on general healthy eating, which particular grains do you recommend? Please select all that apply.
   - Wheat
   - Rye
   - Oats
   - Rice
   - Quinoa
   - Barley
   - Buckwheat
   - Whole grain
   - High fibre grains
   - Other (please specify): ___________________
   - I don't recommend any specific type of grain.
   - I don't recommend any grains.
13. Considering your advice on general healthy eating, which particular grain-based foods do you recommend? Please select all that apply.
   - Breakfast cereal
   - Bread/toast
   - Rice
   - Pasta
   - Crackers
   - Muesli bars
   - Other (please specify): ___________________
   - I don't recommend any grain-based foods.
14. List the grain foods or types of grains you will not recommend.

(open text box)

Whole Grain Foods

The next part of the survey is specifically about whole grain foods.

15. Do you recommend whole grain foods?
   - Yes
   - No
16. Considering your advice on general healthy eating, how often do you recommend whole grain foods in dietetic practice?
   - Always

- Most of the time
- About half the time
- Sometimes
- Never

17. What words might you use in practice to recommend whole grain foods? Please select all that apply.
    - Choose whole grain varieties of bread, breakfast cereal, rice and pasta
    - A quantified suggestion (e.g. choose three of these per day)
    - A specific suggestion (e.g. swap refined ready to eat cereal for oats; swap white for wholemeal/ wholegrain bread)
    - Other (please specify): ________________________
18. What sources of information do you most often use for your advice relating to whole grain food intake? Please select up to two responses.
    - National Dietary Guidelines
    - Government resources
    - Resources from professional associations (nutrition & dietetics)
    - Resources from non-government organisations (e.g. not-for-profit groups promoting whole grain foods)
    - Other (please specify): ________________________
19. In your opinion, what are the nutrition and health benefits of whole grain foods? Please select all that apply.
    - High fibre
    - Low GI
    - Improves weight control
    - Improves blood glucose control
    - Reduces insulin resistance
    - Increases HDL-cholesterol
    - Decreases LDL-cholesterol
    - Lowers blood pressure
    - Reduces inflammation
    - Reduces risk of heart disease
    - Management and reduced risk of type 2 diabetes
    - Protective factor against colorectal cancer
    - Other (please specify): ____________________
20. Typically, in what situations might you prioritise whole grain foods in dietetic practice? Please select all that apply.
    - I don't prioritise whole grain foods in dietetic practice
    - In general dietary advice
    - To increase dietary fibre intake
    - For weight control
    - For diabetes management
    - For blood glucose control
    - For cholesterol management
    - For blood pressure management
    - Other (please specify): ____________________
21. Are there any reasons why you would not recommend whole grain foods to a patient/client/group? *(open text box)*
22. On a scale of 1 to 5, how confident are you in educating clients about whole grain foods? (1 representing not at all confident, 5 representing very confident).

(sliding scale)

23. In your opinion, are there any barriers to whole grain consumption?

(If 'No', skips to Q28)

- Yes
- No
- Unsure

24. In your opinion, what are the barriers to whole grain food consumption? Please select all that apply.
    - There are no barriers preventing whole grain food consumption
    - Culinary skills (e.g. easy recipes)
    - Taste
    - Price
    - Time taken to prepare
    - Availability
    - Concerns about carbohydrate intake
    - Other (please specify): ____________________
25. In your opinion, what strategies could help overcome the barriers to whole grain consumption?

(open text box)

26. Here are some strategies that have been used previously to promote whole grain intake. Please rank which of the following would have the greatest effect (1 being most effective and 6 being least effective) (drag and drop to reorder):
    - Different wording in Dietary Guidelines
    - Promotion via recipes
    - Promotion via media
    - Promotion via front-of-pack labelling
    - Promotion of health benefits
    - Highlighting products through improved front-of-pack scoring systems (e.g. Nutri-Score, Health Star Rating, Traffic Light Guide)
27. Do you believe other dietitians regularly promote and prioritise intake of whole grain foods in practice?
    - Yes
    - Somewhat
    - No
    - Unsure
28. Please indicate the extent you agree or disagree with the following statement. 'Dietitians are well educated about the importance and benefits associated with whole grain food consumption'.
    - Strongly agree
    - Agree
    - Neither agree nor disagree
    - Disagree
    - Strongly disagree
29. How do you think whole grain education for dietitians could be improved? Please select all that apply.
    - Education for dietitians does not need to be improved
    - Online learning / Continuing Professional Development (CPD)
    - Better resources for clients
    - Marketing campaigns
    - Better resources from Dietary Guidelines or National Policy
    - Other (please specify): ____________________
30. Please indicate the extent you agree with the following statement. 'The public is well educated on the importance and benefits associated with whole grain food consumption'.
    - Strongly agree
    - Agree
    - Neither agree nor disagree
    - Disagree
    - Strongly disagree

NOVA Food Classification System

The next part of the survey is about the NOVA food classification system. NOVA was developed in Brazil by Monteiro (2016) and colleagues. It categorises food products into four categories based on their degree of processing – 1. unprocessed and minimally processed foods; 2. processed culinary ingredients; 3. processed foods; 4. ultra-processed foods. Monteiro and colleagues propose that high consumption of ultra-processed foods has negative health impacts. Cross-sectional analysis has linked consumption of ultra-processed foods with risk factors and development of chronic diseases.

31. Prior to reading the description above, were you familiar with the NOVA food classification system?
    - o Yes
    - o No
    - o Somewhat
32. Are you familiar with general advice to limit intake of highly processed/ultra-processed foods?
    - o Yes
    - o No
    - o Somewhat
33. Do you incorporate and/or refer to NOVA or processing of foods in dietetic practice (that is, in consultations, group sessions or media)?
    - o Always
    - o Most of the time
    - o About half the time
    - o Sometimes
    - o Never
34. Do you agree with NOVA's classification of foods?
    - o Strongly agree
    - o Agree
    - o Neither agree nor disagree / not sure
    - o Disagree
    - o Strongly disagree
35. Please elaborate on why you selected the above option:

(open text box)

36. When hearing the term "ultra-processed foods", what foods come to mind?

(open text box)

37. Please indicate which foods you consider to be whole grain and which foods you consider to be "ultra-processed"? **(**foods can be whole grain, ultra-processed or both)

	**I consider this a whole grain food.**	**I consider this a "ultra-processed food".**
Wholemeal bread	Yes/No/Unsure	Yes/No/Unsure
White bread	Yes/No/Unsure	Yes/No/Unsure
Multi-grain bread	Yes/No/Unsure	Yes/No/Unsure
Sourdough bread	Yes/No/Unsure	Yes/No/Unsure
Rye bread	Yes/No/Unsure	Yes/No/Unsure
Porridge oats made from raw oats	Yes/No/Unsure	Yes/No/Unsure
Quick cook oats	Yes/No/Unsure	Yes/No/Unsure
Muesli	Yes/No/Unsure	Yes/No/Unsure
Granola	Yes/No/Unsure	Yes/No/Unsure
Whole wheat cereal breakfast biscuit	Yes/No/Unsure	Yes/No/Unsure
Wheat Bran cereal	Yes/No/Unsure	Yes/No/Unsure
Puffed rice/wheat cereal	Yes/No/Unsure	Yes/No/Unsure
Brown rice	Yes/No/Unsure	Yes/No/Unsure
White rice	Yes/No/Unsure	Yes/No/Unsure
Pasta	Yes/No/Unsure	Yes/No/Unsure
Wholemeal pasta	Yes/No/Unsure	Yes/No/Unsure
Popcorn	Yes/No/Unsure	Yes/No/Unsure

38. Packaged breads (all breads packed in a plastic bag) are classified within NOVA as ultra-processed. Were you aware of this prior to this survey?
    - o Yes
    - o No
39. Ready-to-eat breakfast cereals (all fortified breakfast cereals) are classified within NOVA as ultra-processed. Were you aware of this prior to this survey?
    - o Yes
    - o No
40. Now that you are aware NOVA classifies some whole grain breads and cereals as "ultra-processed foods", please indicate to what extent you agree with the following statements.

I agree with the classification in NOVA for breads as "ultra-processed foods" if they are packaged and fortified

- o Strongly disagree
- o Disagree
- o Neither agree nor disagree
- o Agree
- o Strongly agree
- o I'm not sure

I agree with the classification in NOVA for ready-to-eat breakfast cereals as "ultra-processed foods" even if they are fortified.

- o Strongly disagree
- o Disagree
- o Neither agree nor disagree
- o Agree
- o Strongly agree
- o I'm not sure

Knowing that some whole grain breads and ready-to-eat cereals are classified as "ultra-processed foods" has negatively impacted my perception of these sources of whole grains.

- o Strongly disagree
- o Disagree
- o Neither agree nor disagree
- o Agree
- o Strongly agree
- o I'm not sure

I am less inclined to recommend whole grain breads and ready-to-eat cereals in dietetic practice knowing that they are classified as "ultra-processed foods".

- o Strongly disagree
- o Disagree
- o Neither agree nor disagree
- o Agree
- o Strongly agree
- o I'm not sure

I am less inclined to recommend avoidance of ultra-processed foods knowing that they may include some whole grain foods (such as some ready-to-eat cereals and breads).

- o Strongly disagree
- o Disagree
- o Neither agree nor disagree
- o Agree
- o Strongly agree
- o I'm not sure

I generally agree to avoid ultra-processed foods but do not agree that whole grain breads and cereals should be included in this classification

- o Strongly disagree
- o Disagree

- Neither agree nor disagree
- Agree
- Strongly agree
- I’m not sure

41. Do you have any other comments regarding the NOVA classification system?

(open text box)
