# Supplementary material for: Dietitians’ Attitudes and Understanding of the Promotion of Grains, Whole Grains, and Ultra-Processed Foods
Source: Nutrients. 2022 Jul 23;14(15):3026. doi: 10.3390/nu14153026 (PMC9331638; doi:10.3390/nu14153026)
Supplement: Supplementary file 1 [file nutrients-14-03026-s001.zip › Supplementary Materials 2.pdf]

Supplementary Materials

Table S1. Application of survey questions relative to the constituents of the Theory of Planned Behaviour.

Question	Attitudes	Subjective normative beliefs	Perceived behavioural control
Q10 - Do you recommend or discuss grain foods in consultation, groups sessions or via media messages?	✓		✓
Q11 - Do you promote amounts of grain foods based on National Dietary Guidelines?	✓		✓
Q12 - Are grain foods prioritised in your advice for general healthy eating?	✓		✓
Q13 - Considering your advice on general healthy eating, which particular grains do you recommend? Please select all that apply.	✓		✓
Q14 - Considering your advice on general healthy eating, which particular grain-based foods do you recommend? Please select all that apply.	✓		✓
Q15 - List the grain foods or types of grain you will not recommend.	✓		✓
Q16 - Do you recommend whole grain foods?	✓		✓
Q17 - Considering your advice on general healthy eating, how often do you recommend whole grain foods in dietetic practice?	✓		✓
Q18 - What words might you use in practice to recommend whole grain foods? Please select all that apply.	✓		✓
Q19 - What sources of information do you most often use for your advice relating to whole grain food intake? Please select up to two responses.	✓		✓
Q20 - In your opinion, what are the nutrition and health benefits of whole grain foods? Please select all that apply.	✓		✓
Q21 - Typically, in what situations might you prioritise whole grain foods in dietetic practice? Please select all that apply.	✓		✓
Q22 - Are there any reasons why you would not recommend whole grain foods to a patient/client/group?	✓	✓	✓
Q23 - On a scale of 1 to 5, how confident are you in educating clients about whole grain foods (1 representing not at all confident, 5 representing very confident).			✓
Q24 - In your opinion, are there any barriers to whole grain consumption?	✓	✓	✓
Q25 - In your opinion, what are the barriers to whole grain food consumption? Please select all that apply.	✓	✓	✓
Q26 - In your opinion, what strategies could help overcome the barriers to whole grain consumption?	✓	✓	✓
Q27 - Here are some strategies that have been used previously to promote whole grain intake. Please rank which of the following would have the greatest effect (1 being most effective and 6 being least effective) (drag and drop to reorder):	✓	✓	✓
Q28 - Do you believe other dietitians regularly promote and prioritise intake of whole grain foods in practice?		✓	
Q29 - Please indicate the extent you agree or disagree with the following statement. 'Dietitians are well educated about the importance and benefits associated with whole grain food consumption'.	✓	✓	✓
Q30 - How do you think whole grain education for dietitians could be improved? Please select all that apply.	✓	✓	✓
Q31 - Please indicate the extent you agree or disagree with the following statement. 'The public is well educated on the importance and benefits associated with whole grain food consumption'.	✓	✓	✓
Q32 - Prior to reading the description above, were you familiar with the NOVA food classification system?			✓
Q33 - Are you familiar with general advice to limit intake of highly processed/ultra- processed foods?			✓

Q34 - Do you incorporate and/or refer to NOVA or processing of foods in dietetic practice (that is in consultations, group sessions or media)?	✓		✓
Q35 - Do you agree with NOVA's classification of foods?	✓		
Q36 - Please elaborate on why you selected the above option.	✓	✓	✓
Q37 - When hearing the term "ultra-processed foods", what foods come to mind?	✓	✓	✓
Q38#1 - Please indicate which foods you consider to be whole grain and which foods you consider to be "ul... - I consider this a whole grain food	✓		✓
Q38#2 - Please indicate which foods you consider to be whole grain and which foods you consider to be "ul... - I consider this a "ultra-processed food"	✓		✓
Q39 - Packaged breads (all breads packed in a plastic bag) are classified within NOVA as ultra-processed. Were you aware of this prior to this survey?			✓
Q40 - Ready-to-eat breakfast cereals (all fortified breakfast cereals) are classified within NOVA as ultra-processed. Were you aware of this prior to this survey?			✓
Q41 - Now that you are aware NOVA classifies some whole grain breads and cereals as "ultra-processed foods", please indicate to what extent you agree or disagree with the following statements...	✓	✓	
Q42 - Do you have any other comments regarding the NOVA classification system?	✓	✓	✓
